# Outcome context-dependence is not WEIRD: Comparing reinforcement- and description-based economic preferences worldwide

**DOI:** 10.21203/rs.3.rs-2621222/v1

**Published:** 2023-03-02

**Authors:** Hernán Anlló, Sophie Bavard, FatimaZzahra Benmarrakchi, Darla Bonagura, Fabien Cerrotti, Mirona Cicue, Maelle Gueguen, Eugenio José Guzmán, Dzerassa Kadieva, Maiko Kobayashi, Gafari Lukumon, Marco Sartorio, Jiong Yang, Oksana Zinchenko, Bahador Bahrami, Jaime Silva Concha, Uri Hertz, Anna B. Konova, Jian Li, Cathal O’Madagain, Joaquin Navajas, Gabriel Reyes, Atiye Sarabi-Jamab, Anna Shestakova, Bhasi Sukumaran, Katsumi Watanabe, Stefano Palminteri

**Affiliations:** 1 Human Reinforcement Learning Team, Laboratory of Cognitive and Computational Neuroscience, ENS-PSL, Paris, France.; 2 Faculty of Science and Engineering, Waseda University, Tokyo, Japan.; 3 Intercultural Cognitive Network.; 4 General Psychology Lab, Hamburg University, Hamburg, Germany.; 5 Department of Psychiatry, University Behavioural Health Care, & the Brain Health Institute, Rutgers University—New Brunswick, Piscataway, USA.; 6 School of Collective Intelligence, Universite Mohammed VI Polytechnique, Rabat, Morocco.; 7 Department of Cognitive Sciences, University of Haifa, Haifa, Israel.; 8 International Laboratory for Social Neurobiology, Institute for Cognitive Neuroscience, HSE University, Moscow, Russia.; 9 School of Psychological and Cognitive Sciences and Beijing Key Laboratory of behaviour and Mental Health, Peking University, Beijing, China.; 10 Facultad de Psicología, Universidad del Desarrollo, Santiago de Chile, Chile.; 11 Laboratorio de Neurociencia, Universidad Torcuato Di Tella, Buenos Aires, Argentina.; 12 Centre for Cognition and Decision Making, Institute for Cognitive Neuroscience, HSE University, Moscow, Russia.; 13 IDG/McGovern Institute for Brain Research, Peking University, Beijing, China.; 14 Escuela de Negocios, Universidad Torcuato Di Tella, Buenos Aires, Argentina.; 15 Consejo Nacional de Investigaciones Cientifícas y Técnicas (CONICET), Argentina.; 16 School of Cognitive Sciences, Institute for Research in Fundamental Sciences (IPM), Tehran, Iran.; 17 Department of Psychology, Ludwig Maximilian University, Munich, Germany.; 18 Department of Clinical Psychology, SRM Medical College Hospital & Research Centre, Chennai, India.

## Abstract

Recent evidence indicates that reward value encoding in humans is highly context-dependent, leading to suboptimal decisions in some cases. But whether this computational constraint on valuation is a shared feature of human cognition remains unknown. To address this question, we studied the behavior of individuals from across 11 countries of markedly different socioeconomic and cultural makeup using an experimental approach that reliably captures context effects in reinforcement learning. Our findings show that all samples presented evidence of similar sensitivity to context. Crucially, suboptimal decisions generated by context manipulation were not explained by risk aversion, as estimated through a separate description-based choice task (i.e., lotteries) consisting of matched decision offers. Conversely, risk aversion significantly differed across countries. Overall, our findings suggest that context-dependent reward value encoding is a hardcoded feature of human cognition, while description-based decision-making is significantly sensitive to cultural factors.

## Introduction

Cross-cultural differences in economic decision-making processes have been investigated in several domains, such as risk preference and behavioural game theory. Although several qualitative features seem to be preserved (such as prospect theory-like preferences and delay discounting^1,2^), evidence has repeatedly shown culturally-driven differences in many decision-making traits^3,4,5^.

To date, efforts to assess the cross-cultural stability of decision-making processes have mainly (if not only) focused on what can be defined as “description-based” paradigms, i.e., using tasks where all the decision-relevant information such as prospective outcomes and their “costs” can be inferred from explicit cues or instructions^6,7,8^.

However, little is known concerning the cross-cultural stability (or the lack thereof) of experience-based decisions, which encompass all situations where the decision-making variables have to be inferred from past experience^9,10^. One prominent conceptual framework to investigate experience-based decision processes is reinforcement learning (RL), whose empirical and experimental foundations span multiple disciplines from neuroscience to artificial intelligence^11^.

The lack of cross-cultural investigation of human RL processes is particularly problematic, given that RL is a pervasive cognitive process, with many important implications for mental health, education and economics^12,13,14,15^. Despite its general adaptive value (seek rewards and avoid punishments) laboratory-based research has illustrated that RL processes in many circumstances deviate from a statistical and normative standpoints^16,17^, Determining whether such RL reinforcement learning biases are cultural artefacts, or rather stable components of human decision processes, can provide additional fundamental hints to understand the computational constraints of bounded rationality^18,19^.

Among several features characterizing human RL, the notion of outcome (or reward) context-dependence has recently risen to prominence^16^. More specifically, a series of studies conducted mostly with Western, Educated, Industrialized and Democratic (WEIRD) populations^20^ have shown that in many RL tasks, participants encode outcomes (i.e., rewards and punishments) in a context-dependent manner^21,22,23,24^. While there may not be a consensus yet concerning the exact functional form of such context-dependency, the available findings overwhelmingly favour the idea that subjective outcomes are calculated relatively, following some form of range normalization^25,26,27^. Such context-dependence-induced rescaling of subjective outcomes is often interpreted as a consequence of efficient information coding in the human brain^28,29^. According to this hypothesis, this feature can be understood as the result of fundamental neurocomputational constraints akin to those observable in perceptual decision-making^30,31,32^. In accordance with this proposal of outcome context-dependence in RL as a form of efficient coding, multiple studies using similar tasks in different species have consistently found evidence of range-value adaptation, which suggests that this may be an evolutionary stable, “hard coded”, principle of brain functioning^37,38^.

One well-known consequence of context-dependence in RL is that, in some cases, it can induce suboptimal decisions^25,26,27^. In particular learning contexts, individuals mistakenly attribute higher subjective values to objectively worse options because of how these options are appraised in relation to the local reward distribution, resulting in choices that fail to maximize reward. If indeed there exists such a fundamental computational constraint in the human brain, the behavioural signatures of context-dependence should be a stable feature of decision-making, and thus persist across different populations and cultures. In the present work, we set out to test this hypothesis by leveraging a task capable of eliciting context-dependent RL behaviours, and deploying it across eleven countries of remarkably different socio-economic and cultural makeup (Argentina, Iran, Russia, Japan, China, India, Israel, Chile, Morocco, France and the United States). This allowed us to test the cross-cultural stability of context-dependent value encoding in human RL, and thus assess for the first time its putative role as a core computational process of experience-based decision-making.

In addition, we also administered to our participants a description-based decision-making task that included the same decision contexts as the RL task. The rationale behind this second task was two-fold. First, it allowed us to determine to which extent choice behavior measured in the RL task can be explained by risk aversion, using standard procedures in behavioural economics. Second, it gave us the opportunity to compare for the first time the variability of experience-based and description-based decision-making processes across countries.

Our results indicate a remarkable similarity in how context effects manifest in decisions from experience and suboptimal choice across countries, consistent with the idea that outcome representation in human RL behaviours may reflect conserved constraints on cognition. Our results also showed that risk aversion inferred from the description-based lottery task could not account for these effects. Interestingly, description-based decisions were also found to be highly variable across countries, further confirming the functional dissociation between the behaviour elicited by the two modalities^6,7,33^. Exploratory analyses using independent socio-economic, cultural and cognitive measures taken from our samples further showed that the origin of cross-country differences in description-based decisions is multifactorial, as previously found for risk and other cognitive domains^5,34,35^. Overall, our results suggest that reinforcement (experience-based) decision processes are much more culturally stable than description-based ones and have important implications for theories of bounded rationality^18,19^. We conclude this work by discussing the possible implications of these results for the current implementation of policies and interventions aimed at contrasting the burden of biased decision-making.

## Results

### Behavioural protocol

**Figure 1:**
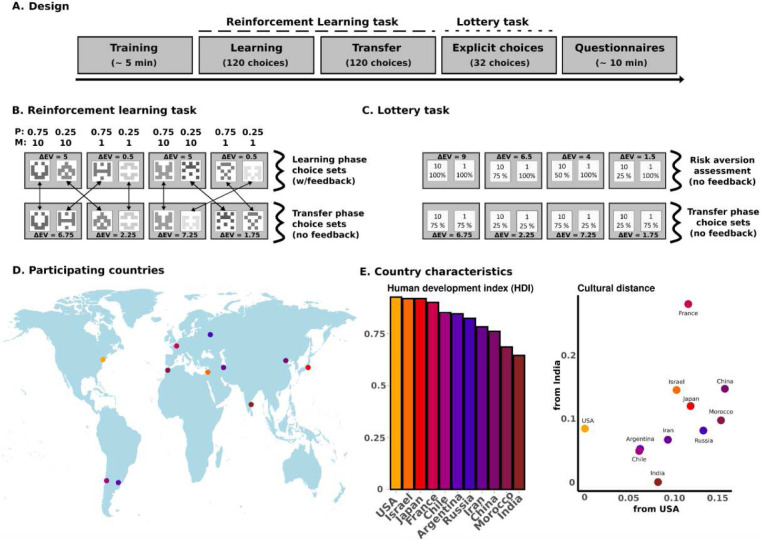
Behavioural protocol and sample. **A. Design.** Outline of the experimental design, including training, RL task, Lottery task and questionnaires. **B. Reinforcement learning task.** Probabilities and magnitudes of each of the lotteries for the Learning and Transfer phases, together with difference in expected value between options for each local decision context. Complete feedback was provided during the Learning phase (factual and counterfactual feedback); no feedback was provided during the Transfer phase. **C. Lottery task**. Probabilities and magnitudes of each of the lotteries for the Lottery task, together with difference in expected value between options for each local decision context. No feedback was provided. **D. Participating countries**. Geographical location of the samples. Dots are placed on the city where data collection was conducted (New Jersey, Haifa, Tokyo, Paris, Santiago de Chile, Buenos Aires, Moscow, Tehran, Beijing, Rabat, Chennai), color-coded as a function of their country’s Human Development Index scores (see panel E - *right)*. **E. Country macrometric characteristics**. Human Development Index scores per country *(left)*, and cultural distance between each country, India and the US *(right)*.

Our behavioural protocol consisted of a reinforcement learning (RL; i.e. experience-based) task, in the form of a previously validated two-armed bandit task^26^, followed by a description-based decision-making task consisting of choices between lotteries (Fig. 1A). Both decision-making tasks were preceded by dedicated instructions and a short training session, and succeeded by a series of questionnaires directed at obtaining information on participants’ socioeconomic, cultural, and cognitive features, as well as general demographics (***Supplementary Materials* - Fig S1**). The RL task consisted of two phases: a Learning phase and a Transfer phase. Its design and implementation reproduced that of Bavard et al., 2021^26^. During the Learning phase, participants were presented with eight abstract icon cues, each representing a lottery of non-disclosed expected value, paired in four stable decision contexts. In the Learning phase, each decision context featured only two possible outcomes: either 10/0 points or 1/0 points. The outcomes were probabilistic (75% or 25%). For convenience, contexts were labelled by taking into account the difference in expected value between the most and the least rewarding option, i.e. the expected value-maximizing (“correct”) and the expected value-minimizing (“wrong”) options (Fig. 1B). In the ensuing Transfer phase, these same eight lotteries were rearranged into new decision contexts [as previously done in similar designs for humans and birds^22,26,36,37,38^]. In addition to the change in decision contexts, the key difference between the Learning and the Transfer phases was that, while during the former participants were presented with complete feedback, in the latter no feedback was provided, so that choices could only be based on values learned during the Learning phase (Fig. 1B). Finally, we conducted an additional task, which we identified as the Lottery task (Fig. 1C). There, the values (magnitudes and probabilities) of the options were explicitly disclosed. The Lottery task featured the same decision contexts used in the Transfer phase, and four additional contexts designed to better assess risk preferences. These last contexts consisted of choices comparing varying probabilities of winning 10 points (100%, 75%, 50%, 25%) against the certainty of winning 1 point.

### Population Demographics

Our main goal was to test the replicability of context-dependence in RL across countries (while disentangling it from risk aversion as standardly assessed in economic value-based decision-making tasks). Thus, our final sample included 11 countries (USA, Israel, Japan, France, Chile, Argentina, Russia, Iran, China, Morocco, India), covering a total of 5 continents and 10 languages (Fig. 1D). Country selection was aimed at portraying a gradual spread across the United Nations’ Human Development Index^39^. This coefficient is built with many metrics, such as GDP, industrialization, mean education level, income inequality, and liberty indexes (Fig. 1E**, *left***). To assess the cultural spread of the selected countries, we used the 1981–2014 dataset of Muthukrishna and colleagues’ cultural distance metric^40^, to estimate the cultural difference between each of the selected countries with respect to the USA and India, which represented the higher and lower HDI values in our sample (Fig. 1E**, *right***).

In order to ensure that our samples would adequately represent the culture of the country to which they belonged, inclusion criteria required that participants: (1) had the target country nationality, (2) resided in the target country, (3) had completed at least the full basic education cycle in the target country, and (4) spoke the country's official language as their native language. These criteria were assessed for each participant during a video meeting prior to launching the experiment. The meeting, task instructions, and questionnaires were delivered in each country's official language, by local researchers.

Additionally, to confirm the diversity of the sample beyond country macrometrics, participants completed individual questionnaires on socioeconomic status^41^, individualistic/collectivistic tendencies^42^, centrality of religiosity in their social environment^43^, and a cognitive reflection test^44^ (see Methods for a detailed description of each metric).

Sample sizes for each country were set based on a power analysis conducted based on the online results of Bavard et al., 2021^26^ (n = 46 per country, see Methods). After exclusions (failure to complete the task n = 43; troubleshooting/translation issues during task rollout n = 19), a remainder of n = 561 participants (342 female; mean age(SD) = 24.4(4.6)) composed the final sample (n = 51 on average per country). Separate linear regressions, using each of the demographic and sociocultural indexes as predictors of nationality, confirmed that country samples were significantly different in many respects. A summary of these differences, demographic information, sample sizes and exclusions can be found in Table 1. Detailed results of the regressions can be found in the Supplementary Materials (**Table S1**).

### Reinforcement learning task (experience-based)

We first looked at performance in both RL phases. We focused on correct responses (i.e., probability of picking the expected value-maximizing choice) as the behavioural dependent variable. Correct response rate was analysed separately in each RL phase (i.e. Learning and Transfer), as a function of decision context (within-subjects variable) and country (between-subjects variable). We also compared the correct response rate against chance level (0.5) to assess learning and preferences. As in previous studies using the same or similar designs^22,26^, of particular relevance for the demonstration of outcome context-dependence were: i) the comparison of accuracies between the ΔEV = 5.0 and the ΔEV = 0.5 decision contexts in the Learning phase (where absence of difference – magnitude effect - is taken as a sign of relative value learning) and ii) the preference expressed in the ΔEV = 1.75 decision context of the Transfer test (where below-chance accuracy is taken as an indicator of context-dependent value rescaling).

**Figure 2:**
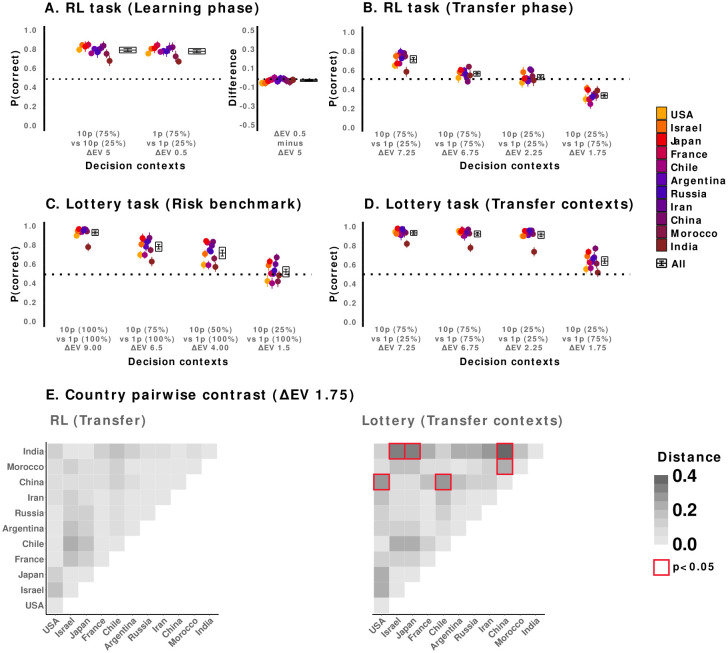
Behavioural results. **A. RL task (Learning phase**). Proportion of correct answers (i.e. choices that maximize expected value) for each individual country (dots) and the average of all countries (box) for each of the two decision contexts of the Learning phase. **B. RL task (Transfer phase**). Proportion of correct answers (i.e. choices that maximize expected value) for each individual country (dots) and the average of all countries (box) for each of the four decision contexts of the Transfer phase (leftmost part). Difference between the big (ΔEV=5.0) and the small (ΔEV=0.5)magnitude context (rightmost panel) C. **Lottery task (benchmark of risk preferences**). Proportion of correct answers (i.e. choices that maximize expected value) for each individual country (dots) and the average of all countries (box) for each of the four decision contexts of the Lottery task presented to estimate risk aversion. **D. Lottery task (Transfer decision contexts**). Proportion of correct answers (i.e. choices that maximize expected value) for each individual country (dots) and the average of all countries (box) for each of the four decision contexts of the Lottery task that were homologous to the decision contexts of the Transfer phase. **E. Country pairwise contrasts for the ΔEV = 1.75 decision context.** Euclidean distance between mean proportion of correct answers of each country during the RL task (left). Euclidean distance between mean proportion of correct answers of each country during the Lottery task (right). *Bars represent standard error of the mean. Midline of box represents mean of all countries. Bounds of box represent the 95% confidence interval of the mean. Red boxes represent a significant pairwise contrast.*

Results showed that the average correct response rate for the Learning phase was significantly different from chance level 0.5 for all countries and decision contexts (Fig. 2A), which confirmed that learning had occurred (pooled sample: ΔEV = 5, 0.8 ± 0.2, t(560) = 42, p < .0001, d(95% CI) = 1.8(1.66, 1.92); ΔEV = 0.5, 0.8 ± 0.2, t(560) = 38, p < .0001, d = 1.6(1.49, 1.74); see ***Supplementary Materials*** - **Table S3** for model selection, **Table S4** for full regression results). While we found significant differences in aggregate performance between countries (Country main effect: χ2 = 58, DF = 10, p = <.0001), learning and above-chance performance levels were observable in all samples and contexts (**Fig. S2**).

Importantly, we did not find evidence for any magnitude effects in any of the country samples, meaning that the learning performance was the same in the ΔEV = 5 and the ΔEV = 0.5 in all countries (Decision context main effect: χ2 = 2, DF = 1, p = 0.14; Decision context × Country interaction: χ2 = 12, DF = 10, p = 0.29). Further AICc weight ratio analysis confirmed a lack of magnitude effect (i.e., a model including Decision Context as a regressor was 0.01 times as likely to predict accuracy as the same model without it).

We then turned to the analysis of the Transfer phase (Fig. 2B). In this case, correct choice rates were strongly modulated across decision contexts (Decision Context main effect: χ2 = 326, DF = 3, p = <.0001). Here, we did not find evidence for any country effects (Country main effect: χ2 = 18, DF = 10, p = 0.05; Decision context × Country interaction: χ2 = 41, DF = 30, p = 0.09). Further AICc weight ratio analysis indicated a lack of Country effect (i.e. a model including Country as a regressor was 0 times as likely to predict accuracy as the same model without it).

Replicating previous findings, and indicating that participants could successfully retrieve and generalize the values learned during the Learning phase, correct choice rates in the ΔEV = 7.25 and the ΔEV = 6.75 decision contexts were well above chance level (0.7 ± 0.3, t(560) = 15, p < .0001, d = 0.6(0.55, 0.73); ΔEV = 6.75, 0.56 ± 0.4, t(560) = 3.5, p < .001, d = 0.15(0.07, 0.23)). Crucially, however, accuracy in the ΔEV = 1.75 context was below chance level for all countries, indicative of context-dependence induced suboptimal preferences (pooled sample: 0.33 ± 0.3, t(560) = −12, p < .0001, d=−0.5(−0.6, −0.4); see individual per-country T-tests in ***Supplementary Materials* - Table S5**). Once again, while significant differences in aggregate performance between samples were found (Country main effect: χ2 = 19, DF = 10, p = .04), the evidence did not indicate any interaction between country and decision contexts (Country × Decision context interaction: χ2 = 40, DF = 30, p = 0.1). Crucially, the presence of suboptimal behaviour in the ΔEV = 1.75 context was observable in every country (see ***Supplementary Materials* - Table S5**), with no significant differences between countries (Fig 2.E**, *left***; see ***Supplementary Materials* - Table S6** for post-hoc pairwise contrasts).

These results replicated previous findings^22,26^, and showed that the behavioural signatures of outcome context-dependence were cross-culturally stable in the RL task. Contrary to what a model encoding values on an absolute scale would have predicted, performance was not affected by the outcome magnitude during the Learning phase: this constitutes a positive manifestation of context-dependent adaptive coding^28^. Additionally, preferences were globally below chance in the ΔEV = 1.75 condition. Namely, a previously optimal option (EV = 0.75) was preferred to a previously suboptimal option (EV = 2.5) despite its expected value being higher in the new decision context. This illustrated the already-known negative side of outcome context dependence in the context of RL: suboptimal decisions may arise when options are extrapolated from their original context.

### Lottery task (description-based)

We then analysed participants’ preferences in the description-based Lottery task (Figs. 2C, 2D). We first considered choices in the decision problems aimed at benchmarking risk preferences, where a sure small payoff (1pt) was presented against risky options with varying probabilities of delivering a bigger payoff (10pts). These four decision problems allowed us to estimate risk preference, quantified as the decrease in expected value-maximizing choice rates as the probability for obtaining the larger payoff decreased (i.e. propensity to choose the objectively higher value option as the levels of risk for that option increased). Results showed a coherent modulation of decision context on choice behaviour: as the risk involved increased, choice ratios for the objectively higher value offers decreased for all countries (pooled sample: ΔEV = 9, 0.94 ± 0.1, t(560) = 60, p < .0001, d = 2.6; ΔEV = 6.5, 0.79 ± 0.2, t(560) = 23, p < .0001, d = 1; ΔEV = 4, 0.72 ± 0.3, t(560) = 16, p < .0001, d = 1; ΔEV = 1.5, 0.53 ± 0.4, t(560) = 2, p = 0.09, d = 0; Decision Context main effect: χ2 = 326, DF = 3, p = <.0001; see ***Supplementary Materials* - Table S3** for model selection, **Table S4** for full regression results). Interestingly, while risk affected performance for all country samples, it did so differently across countries (Country main effect: χ2 = 57, DF = 10, p = <.0001; Country × Decision Context interaction: χ2 = 100, DF = 30, p = <.0001; see ***Supplementary Materials* - Table S5** for per-country T-test analyses). This indicated that preferences expressed in the description-based task were not cross-culturally stable, unlike behaviour observed in the RL task.

After assessing the detectability of risk aversion in the benchmark decision contexts of the Lottery task, we analysed preferences in the decision contexts homologous to those of the Transfer phase in RL (Fig. 2D). This allowed us to directly compare between experience-based and description-based preferences. We focused mainly on the behaviour expressed at the ΔEV = 1.75 decision context, where a tendency to significantly choose suboptimal choices can be interpreted as a sign of context dependence in the RL task. Crucially, and contrary to RL behavior, results showed that in all countries correct choice rate was significantly above chance for this decision problem in the description-based task (pooled sample: ΔEV = 7.25, 0.9 ± 0.1, t(560) = 58, p < .0001, d = 2.4; ΔEV = 6.75, 0.9 ± 0.1, t(560) = 51, p < .0001, d = 2; ΔEV = 2.25, 0.9 ± 0.1, t(560) = 47, p < .0001, d = 2; ΔEV = 1.75 0.6± 0.4, t(560)=9, p<.0001, d = 0.4). Additionally, the ΔEV = 1.75 Lottery context presented evidence of significant between-country differences, absent in RL (Fig 2.E**, *right***; Country × Decision Context interaction: χ2 = 68, DF = 30, p = <.0001, see ***Supplementary Materials* - Table S6** for post-hoc pairwise contrasts). In order to directly compare between descriptive and experiential choices at the ΔEV = 1.75 context, we modelled preferences in this decision context by including an additional regressor (Decision Type; levels: RL, Lottery). Results indicated a significant Decision Modality effect (χ2 = 216, DF = 1, p = <.0001) that confirmed the difference between the two tasks.

Overall, results from the Lottery task illustrated two important points. First, we were able to detect significant across country behavioural differences in our sample. This excludes that absence of effect in the RL task can thus not be ascribed to a general inability of detecting behavioural differences with our protocol. Second, these findings showed that risk aversion, as inferred from preferences expressed in the Lottery task, could not account for preferences in the RL task. This was specifically true for the key ΔEV=1.75 decision context, where we observed a clear case of preference reversal when comparing the two decision modalities^45^.

### Computational results

To quantify the observed decision-making strategies in a systematic manner that encompassed all decision contexts across all tasks, we formalized choice behaviour using simple models built around the notion of subjective outcome scaling. This choice was motivated by the fact that this outcome scaling process, described below, could satisfactorily and parsimoniously capture the behavioural consequences of both context-dependent outcomes (in RL) and decreasing marginal utility (in Lottery). In both tasks, the subjective value of a given outcome or payoff was adjusted through the implementation of a free parameter (0 ≤ *v* ≤ 1) as follows:

$$R_{\text{scaled},t}=\{\begin{array}{cc} 10p*v,\;\text{if} & R_{obj,t}=10p\\ R_{obj,t} & \;\text{otherwise} \end{array}$$

where R_*scaled*,t_ represented the scaled subjective outcome and R_*obj,t*_ the objective unscaled outcome at trial *t*. For RL trials, we embedded the scaling process within a fully-parameterized version of the standard Q-learning algorithm, where option-dependent Q-values were learnt from the range-adapted reward term R_*scaled*_. The algorithm also included free “temperature” [*β*], “forgetfulness” [*ϕ*] and “learning rate”[*α*] parameters, inasmuch as the RL process consists of acquiring value from experience and subsequently storing those values in memory for value actualization and learning^11^. For the Lottery task trials, we formalized choice behaviour based on the subjective expected value that participants attributed to each choice as a function of its inherent risk, by multiplying R_*scaled,t*_ by reward probability (as customarily done in standard linear utility models^46^). While we did retain choice temperature [*β*] for this instance of the model, no memory actualization or learning processes were expected to take place during Lottery, which rendered *ϕ* and *α* unnecessary. We differentiated between scaling and temperature in RL and Lottery decision contexts by fitting specific parameters as *v*_RL_, *β*_RL_ and v_LOT_, *β*_LOT_, respectively. We made sure that our fitting procedure allowed us to correctly recover the parameters in simulated datasets, as well as produce simulations that would closely replicate the observed behavioural data (see ***Supplementary Materials*** for procedure and results of simulations and parameter recovery).

Utilizing the same scaling parameter [*v*] in both models was a crucial step in the formalization, as it allowed us to compare experiential and descriptive adaptation mechanisms in the same terms, while integrating all the possible decision contexts. We expected v_RL_ to reflect context-dependent range-value adaptation in the RL task, and v_LOT_ to capture marginally decreasing utility (and therefore risk aversion) in the Lottery task. It follows that v_RL_ was expected to remain invariant across country samples, confirming that relative value-encoding occurred universally, and independently of risk preferences. Conversely, we expected v_LOT_ to differ significantly between countries, in line with the observed risk aversion behaviours for each country sample, and to be decorrelated from v_RL_.

**Figure 3.**
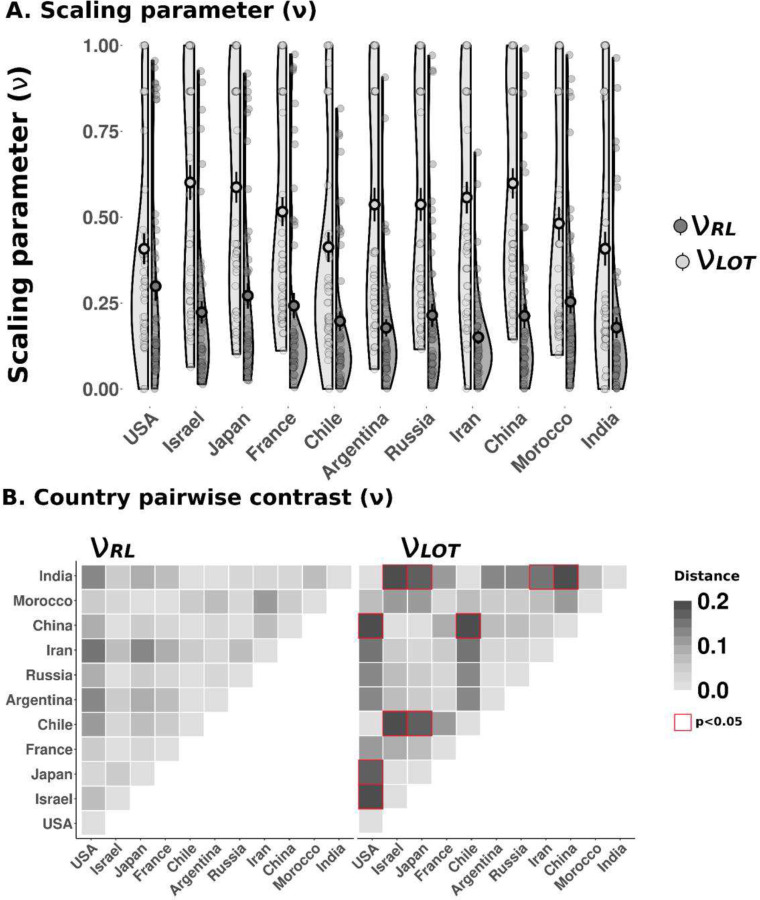
Computational results. **A. Scaling parameter values**. Values of the scaling free parameter estimated during the RL task (v_RL_) and the Lottery task (v_LOT_). **B. Country pairwise contrasts for the scaling parameters**. Euclidean distance between mean of scaling parameters of each country during the RL task *(left)*. Euclidean distance between mean of scaling parameters of each country during the Lottery task *(right)*. *Translucent dots are individual participants’ values; underscored dots represent the mean, bar represents standard error of the mean. Red boxes represent a significant pairwise contrast.*

As shown in Fig. 3A, scaling patterns conformed to these hypotheses. First, we found minimal evidence for differences between countries in v_RL_ (v_RL_ ~ Country; SS = 0.98, DF = 10, p = 0.07). We confirmed this lack of effect through AICc weight ratio analysis: we considered a full model including Country as a predictor, and as null an identical model not including it. Results strongly disfavoured Country as a relevant predictor of v_RL_ in terms of information loss (i.e. full model having 0.23 times the strength of the null model). Second, evidence showed that v_LOT_ differed significantly across country samples (v_LOT_ ~ Country; SS = 3, DF = 10, p < 0.01). Here, AICc weight ratio strongly favoured the Country effect model (full model being 16.65 times stronger than the null model). Finally, as seen in Fig. 3B, between-country pairwise contrasts revealed significant differences in v_LOT_ (see ***Supplementary Materials* - Table S9** for post-hoc pairwise contrasts). Indeed, v_LOT_ differed substantially across countries, from quite substantial risk aversion (median v_LOT_ = 0.28 in the Chilean sample) to moderate-high (median v_LOT_ = 0.62 in the Israeli sample). Crucially, v_LOT_ values were highly correlated with the risk aversion behavioural patterns previously observed in the ΔEV = 1.5 and ΔEV = 1.75 Lottery trials (R = 0.84 (95% CI = 0.81, 0.86) and p < .0001; R = 0.64 (95% CI = 0.59, 0.69) and p < .0001), and decorrelated from v_RL_ (R = 0.08 (95% CI = 0, 0.16) and p = 0.24) (see ***Supplementary Materials* - Fig. S4, Table S7**).

In sum, our computational approach confirmed strong evidence for stable cross-country outcome context-dependence in the RL task using a compact computational measure. A similar analysis performed in the Lottery task, confirmed that the preferences in the RL task could not be accounted for risk aversion inferred from the Lottery task. Crucially, these results also confirmed a difference in the stability of experience- and description-based processes across countries.

In order to discard that the differences found in scaling between phases could be confounded by differences in task performance (i.e., lack of learning, inattention), we reanalysed and refitted the data after excluding all participants who had less than 100% accuracy in choices involving fully-dominated options in the Lottery task (as seen in previous studies on economic preferences^47,48^). In such contexts (i.e. ΔEV = 7.25 and ΔEV = 9), suboptimal choices can be ascribed to general inattention, or the use of task-irrelevant heuristics (e.g. basing choices on a cue’s visual features, etc). These analyses, available in the Supplementary Materials section, confirmed that this strict elimination criterion improved overall performance (and resulted in less stochastic choices as proxied by the increase of both *β*_RL_ and *β*_LOT_)· However, even after exclusion of these participants (n = 124 Total remaining n = 437), we were still able to replicate all behavioural and computational patterns of results presented thus far (see ***Supplementary Materials* - Figs S5–S8**).

### Drivers of risk aversion differences

Our main goal was to test whether the behavioural and computational signatures of context-dependent outcome encoding in RL would replicate across samples from different countries and cultural backgrounds, and whether or not said preferences would differ from those of a description-based task. We indeed found positive evidence showing that context-dependence as captured in experience-based decision-making tasks is stable across the included countries and distinct from risk aversion in tasks from description. Importantly, we did not have any specific directional prediction on what cultural or socio-economic factors would influence preferences in general (and more specifically, risk aversion in the Lottery task). However, in an exploratory manner, we evaluated if the cultural and socio-economic metrics we had obtained characterized the differences in risk aversion between samples. We did so by producing separate linear regressions of the scaling (v_RL_ and v_LOT_) and temperature (*β*_RL_ and *β*_LOT_) parameters against our country-level and subject-level cultural, economic and cognitive metrics. Results of these exploratory analyses (see ***Supplementary Materials* - Table S12**) showed that single-dimension subjective metrics did not significantly predict the values of the outcome scaling parameters, for either task. On the other hand, country-level macrometrics composed of multiple dimensions (i.e. HDI, Cultural Distance) did improve the models. This fell in line with previous findings on intercultural risk preferences, which show that individual differences rarely inform risk preferences, but country-level macrometric indexes are marginally better^5,34,35^. It should be noted however that even when significant, the correlation magnitudes were considerably small. Nonetheless, it should be noted that cultural metrics generally predicted changes in v_LOT_, but not v_RL_, which was consistent with the robustness of RL biases to cultural factors, as well as the gap between experiential and descriptive choices found in our main results.

## Discussion

In the present work, we sought to assess the cross-cultural stability of a recently discovered but well-documented feature of human behaviour: context-dependent value encoding. It is important to underscore that however robust, the vast majority of the results concerning context effects in human RL come to date from WEIRD samples^16,21,22,23,24,25,26,49^. This severely limits the interpretation of context-dependent value encoding as a fundamental cognitive building block of human choice behaviour in general. Here, we aimed to address this issue by showing marked evidence of outcome context-dependence in samples from 11 countries of different sociocultural makeup. Outcome context-dependence was evident both from behavioural signatures (i.e., magnitude invariant performance in the Learning phase; persistent suboptimal preferences in the Transfer phase), and from the analysis of the key parameter of our computational model (i.e., v_RL_). In addition to our RL task, we also administered a description-based task featuring the same decision contexts. This allowed us to demonstrate for the first time that risk aversion (as standardly inferred in behavioural economics from lottery tasks) could not account for behavioural signatures of context-dependence in the RL task (especially suboptimal preferences). Further, we have also shown that while experience-based processes and preferences were remarkably stable across the included countries, description-based processes were not.

By replicating the finding of value context-dependence outside the WEIRD space, our work shows that this cognitive process is not likely to be a simple cultural artefact^50,51^. Of course, we acknowledge that our current sample is not diverse enough to argue for a *definitive* universality of contextual value encoding in RL. We also acknowledge that our samples may be neglecting within-country variations (some of the included countries contain within themselves very different ethnic and linguistic communities that we did not cover). However, the fact that our results would show this bias consistently throughout samples constitutes strong evidence in that direction, particularly since our samples were distinct enough to elicit between-country differences in explicit value-based choices. Future research efforts seeking to extend the present findings should consider testing in rural vs urban population setting^52^, and across different social layers within the same societies^2^.

The presence of context-dependent value learning across such a diverse sample falls in line with numerous prior findings pointing to the reliability of the phenomenon. Multiple studies have shown the flexibility of context dependence across different contexts^36^, its validity for non-binary outcomes^24^ and non-binary decision spaces^53^, and different temporal learning dynamics^54^. Furthermore, instances of context-dependent value learning have also been observed reliably in a wide range of non-human animals, as diverse as mammals, birds and insects^38,55^. The coincidence between our present cross-cultural results and the ample array of cross-species prior findings, reinforces the notion that RL processes may be largely hard-coded and evolutionary stable^56^. Indeed, despite the incidental generation of suboptimal preferences (e.g., in the Transfer phase), context-dependent value learning likely presents an overall adaptive value. Theoretical propositions suggest that the normativity of context-dependent value learning can be traced to at least two, not mutually-exclusive sources. First, it is possible that outcome-context dependence in RL may constitute just another manifestation of the adaptive coding phenomenon^28,29^. In adaptive coding theory, the neural representations of objective variables are transformed as a function of their underlying distribution, as a means to adjust to neural constraints in information processing^30,57,58^. Second, it is also possible that context-dependent value learning serves the purpose of maximizing performance (i.e., “fitness”) in many ecological foraging situations^59^. Namely, encoding the *convenience* of a choice with respect to its alternatives in context (i.e. storing the result of a computation rather than all of its components) would be much less resource-intensive and ecological than committing to memory large repertoires of absolute values dissociated from their contexts^60^.

A crucial contribution of the present work is the analysis of behavioural performance in a description-based decision-making task featuring the same decision problems as in the Transfer phase (in addition to other benchmark decision problems). This allowed us, first and foremost, to rule out the possibility that an absence of cross-cultural variation in context-dependent value learning could be merely due to our inability to detect *any* cross-cultural differences in choice behaviour in our sample. This was not the case, as we observed that behavioural preferences elicited during the Lottery task were significantly different across countries, and in line with each sample’s risk preferences. As with previous cross-cultural studies on decision-making, differences in lottery-elicited risk preferences were found to be multicausal^5,34,35^. Possible causes for this lack of clarity in the etiology of risk preferences can be traced to the diversity of methods used to quantify risk aversion across studies, and to the fact that most of the tested predictors evaluated so far have been shown to account for only small fractions of the total variance^35^. As stated, pinpointing the cultural drivers of differences in risk preferences across countries was beyond the scope of the present work. Given their effect size and exploratory nature, these results can not be interpreted at the moment as anything more than venues for future research. Still, our findings highlight the necessity of developing a unified strategy for quantifying risk preferences, that may take into account the socio-economic, demographic and cognitive characteristics of intercultural samples^61^.

Importantly, the addition of an explicit set of decision problems homologous to those of the RL task allowed us to compare experience-based and description-based choice behaviour. This led us to show, to the best of our knowledge for the very first time, that in otherwise comparable decision contexts, risk aversion as inferred from a standard lottery task does not explain preferences in the Transfer phase of a RL task. This was particularly noteworthy for the ΔEV = 1.75 decision context, in which suboptimal choice preferences are customarily considered a hallmark of context-dependence in value learning^23,26,38^. Indeed, in the present work, preference reversal in this context was observable for all countries during RL, and shown to be different from risk-driven choice behaviour, thus calling for an alternative explanation.

These differences between the RL and Lottery tasks, concerning both subjective outcome encoding and cross-cultural stability, were well recapitulated by our modelling approach. We devised a simple parsimonious outcome-scaling process, that fitted to both experiential and described versions of our decision problems, leading to the emergence of two clearly distinguishable sets of values for the scaling parameter. It is important to underscore that, while for parsimony and commensurability purposes, we modelled preferences in RL and Lottery tasks with the same outcome-scaling model, this does not imply the assumption that both tasks share similar computational processes. Indeed, based on the present and other behavioural findings^13,21,26^ it is likely these different value scaling schemes arise from different underlying computations altogether, respectively, outcome range-adaptation in RL and diminishing marginal utility in Lottery (see ***Supplementary Materials*** for further considerations). It is nonetheless important to note that here we are not claiming that context-dependent valuation is exclusivity of experience (or reinforcement) based choices. In fact, many contextual effects have been documented in descriptive choices (such as the decoy effect). Further studies should determine whether such effects of description-based choices are cross-culturally stable.

The present results broadly fit within the larger framework of the experience-description gap, by showing that preferences for the same decision problems are strongly affected by the modality in which the problems are presented^6,7,62^. This begs the question of whether or not differences in probability weighting, which are robustly reported between experience-based and description-based decisions, could explain the observed discrepancy, and more specifically, the preference reversal in the ΔEV = 1.75 decision context^8^. *Prima facie*, the fact that the “1 point with 75% chance” option would be preferred to the “10 points at 25% chance” option, is compatible with the traditional experienced-based pattern of underweighting rare events^7,63^. However, it should be noted that for the preference reversal to derive solely from different probability weightings it would require a probability distortion much larger than what has commonly been observed in experiments and meta-analysis to date^8,64^. Furthermore, the Learning phase of our experience-based task featured complete feedback, a manipulation that makes feedback information independent from choice, and thus reduces or even eliminates insufficient probability sampling (which is the traditional explanation for the classical probability weighting of experience-based choices). Finally, the underweighting of rare events would not explain the absence of a magnitude effect during the RL Learning phase. Conversely, outcome context-dependence does provide a satisfactorily and parsimonious explanation for the observed choice patterns in both the Learning and Transfer phases.

Finally, we offer some reflection on the implications of our findings for behavioural science-inspired interventions in policy-making. In recent years, the idea that descriptive models of behavioural decision-making should be used to inform better policies (top-down), or for designing better decision architectures (bottom up) has gained traction^65,66,67^. In the long-term, this approach may help improve both individual and collective decision-making in domains where biases and suboptimal decision-making represent key bottlenecks (e.g., issues such as choice of vaccination, or behaviours favouring environmental protection). Historically, decision models in (behavioural) economics, nudging and behaviourally-inspired policies have been based on description-based choice behaviour. Our results show that, compared to description-based processes, experience-based decision models are much more stable on a cross-cultural level, possibly capturing deep and preserved features of human cognition. We therefore believe that, especially if this pattern is confirmed and generalized to other tasks and processes, the present work calls for a better consideration of experience-based decision models in designing behavioural science-informed public policies in general.

## Methods

### Participants:

Recruitment was conducted locally, through the standard channels of each participating institution (e.g. dedicated mailing lists, flyers and online ads). Sample size was determined through a power analysis based on the behavioural results of Bavard et al., 2021 online experiment^26^. In the ΔEV = 1.75 context of said experiment (blocked trials, complete feedback version), online participants reached a difference between choice rate and chance (0.5) of 0.27 ± 0.30 (mean ± SD). To obtain the same difference with a power of 0.95, the MATLAB function “samsizepwr.m” indicated that 46 participants per country were needed. Samples were allowed to exceed this limit by up to 20%, to ensure the desired power would be achieved regardless of potential participant exclusions. Exclusion criteria consisted of failure to complete the task (n = 43) and troubleshooting/translation issues during the online task rollout (n = 19). A remainder of n = 561 participants (342 female; mean age(SD) = 24.4(4.6)) composed the final sample.

### Ethics:

research was carried out following the principles and guidelines for human experimentation provided in the Declaration of Helsinki (1964, revised in 2013). This study belongs to a series of experiments approved by the INSERM Ethical Review Committee/IRB00003888 on 13 November 2018. Wherever needed, this ethical authorization was seconded with further authorizations at the local level at the behest of each participating institution. All participants provided written informed consent before their inclusion.

### Payment:

To sustain motivation throughout the experiment, participants were given a bonus depending on the number of points won in each task. To ensure motivation would be even across countries, each participating institution calculated the average cost of a local university lunch (inter-country average cost in euros: 5.8 ± 2.82), and divided it by the total amount of points to be potentially won throughout the experiment (i.e. 1275 points for a perfect run; average value of point in euros: 0.0045 ± 0.002; average bonus reward obtained in euros: 5.4±1.53). In addition to the bonus accrued through point accumulation, all participants received a flat participation rate equivalent to an additional student lunch (see ***Supplementary Materials* - Table S2** for average bonuses in local currencies).

### Behavioural task:

there were two behavioural tasks, the Reinforcement Learning (RL) task and the Lottery task (Fig 1.A). The RL task was a direct reproduction of the probabilistic instrumental learning task performed in Experiment 7 of Bavard et al.,, 2021^26^. Participants were asked to choose on a trial basis between the undisclosed lotteries of different 2-armed bandit problems, with the goal of maximizing overall reward. The Lottery task consisted of a standard economic decision-making task, where participants had to choose on a trial basis between two lotteries of known expected value, again with the intention of maximizing overall reward.

In the RL task, the lotteries for each decision context were represented by abstract stimuli (cues) taken from randomly generated identicons. Identicons were generated so that hue and saturation had similar values within the HSLUV colour scheme (www.hsluv.org). In the Lottery task, cue cards displaying the reward and probability values for each option were used instead. For all tasks, each decision context was formed by two cues, one at each side of the screen, equidistant to the screen centre. Each trial consisted of a single decision context. Stimulus location was pseudo-randomized, so that every cue would appear an equal number of times on each side of the screen. In the RL task, participants had to complete a Learning phase, and then a Transfer phase^16,21,22,23,24,25,26,49^. In the Learning phase (Fig. 1B**, *upper****)*, cues appeared in four different fixed pairs (i.e. decision contexts). Within pairs, each cue would lead to possible zero and non-zero outcomes with reciprocal probabilities (0.75/0.25 and 0.25/0.75). Each decision context featured only two possible outcomes: either 10/0 points or 1/0 points. Contexts were labeled by taking into account the difference in expected value between options (i.e., two ΔEV = 5 and two ΔEV = 0.5 decision contexts). Once a choice was made by clicking on a cue, a fixed 500 ms delay ensued, after which factual and counterfactual choice feedback was displayed for 1000 ms in the form of “10,” “1,” or “0” points cue cards. After Learning phase completion, the subtotal of points earned was displayed, together with its monetary equivalent in local currency. In the Transfer phase, cues were rearranged into four new pairs (ΔEV = 7.25, ΔEV = 6.75, ΔEV = 2.25, and ΔEV = 1.75). Crucially, the probability of obtaining a specific outcome from each cue remained the same as in the Learning phase (Fig. 1B, ***lower****)*. In the Lottery task (Fig. 1C), participants had to choose between explicit cue cards, which were paired reproducing the 4 decision contexts of the Transfer phase, and another 4 decision contexts comparing varying probabilities of winning 10 points (100%, 75%, 50%, 25%) versus the certainty of winning 1 point (ΔEV = 9, ΔEV = 6.5, ΔEV = 4, ΔEV = 1.5). Neither Transfer phase nor the Lottery task presented any post-choice feedback: choices were followed by a fixed 500 ms delay interval, after which “???” cue cards were displayed for 1000 ms. Each decision context of the RL task (4 in Learning phase, 4 in Transfer phase) was presented 30 times, for a total of 240 trials. Decision contexts of the Lottery task (4 reproducing Transfer, 4 benchmarking risk aversion) were presented 4 times each, for a total of 32 trials. Presentation order of decision contexts was pseudo-randomized within each phase, so that all trials of a given decision context would be cr lustered (i.e., “blocked” stimuli presentation).

### Questionnaires:

after completing the behavioural experiment, participants were required to complete several psychometric and socioeconomic questionnaires. Socioeconomic questionnaires included the Individualistic and collectivistic tendencies inventory^42^, the perceived Socioeconomic status in childhood, adulthood and social hierarchy questionnaires^41^, and the Centrality of religiosity questionnaire^43^. The sole goal of these questionnaires was to confirm that samples were socioculturally different from each other, as simply belonging to different countries may not have ensured a difference. Psychometric questionnaires were incorporated for purely exploratory purposes, including the Ten Item Personality Inventory (TIPI)^68^ and the extended version of the Cognitive Reflection test (CRT)^44^. Order of questionnaires, and questions within each questionnaire, were randomized (see ***Supplementary Materials*** for a technical description of each questionnaire, and exploratory analyses).

### Country metrics:

questionnaires gave us the opportunity of assessing different dimensions of the socioeconomic and cultural makeup of each country sample from participants’ own subjective answers. To quantify the socioeconomic and cultural profile of each country sample in a macrometric way, we also incorporated into the analysis each country’s Human Development Index score^39^, and the Cultural Distance between countries^40^. Both of these coefficients are computed from combining large numbers of economic, educational, political and psychosocial markers. Under the same rationale as questionnaires, inclusion of these metrics was not hypothesis-driven, but rather served to establish the differences between country samples and conduct exploratory analyses (see ***Supplementary Materials*** for details on metrics).

### Procedure:

Testing was conducted in a hybrid face-to-face/online format, where participants met a local experimenter for an online live debrief held in their local language to verify identity and cultural affiliation. After the interview, participants received a personalized link to a Gorilla server (www.gorilla.sc) where the experiment was hosted. After clicking on the link, participants were sent to a consent form, which they had to complete in order to access the actual experiment. The experiment started by providing written instructions on how to perform the task. It was explained to participants that they would have to choose between two different options over several trials, with the goal of maximizing overall point reward. It was told to them that they would have to make this decision without necessarily knowing the probability and magnitude of rewards for each option at first. Finally, it was explained at length that their final payoff would be affected by their choices, as rewards were convertible to actual currency. The possible outcomes in points (0, 1, and 10 points) were explicitly shown, as well as the point-currency conversion rate for their country (e.g. 1 point = 0.005 euros in France; see ***Supplementary Materials* - Table S2**). Instructions were followed by a short training session of 12 trials, designed to familiarize participants with response modality. Participants could decide to repeat the training session up to two times prior to starting the actual experiment. After finishing the training session, participants had to complete the RL task (Learning and Transfer), the Lottery task and the sociocultural questionnaires, in that order. The existence of the Transfer phase was not disclosed until the end of the Learning phase, to prevent the use of alternative strategies. Crucially, before starting the Transfer phase, participants were made explicitly aware of the fact that they would be presented with the same cues they had seen during the Learning phase, but combined in different pairs. Before starting the Lottery task, participants were shown an example of a cue card with its explicit reward probability and magnitude written on it, and were again instructed to choose the option that they thought would maximize overall point reward. Following completion of the Lottery task, participants had to answer all sociocultural and psychometric questionnaires. Order of questionnaires as well as order of each item within questionnaires were randomized. Completing the full experiment, including consent and questionnaires, took approximately 25 minutes (average response time per trial 1.46 ± 6.7 s; median 0.96 s). Once finished with the experiment, participants were given a personalized completion code, and were tasked with sending this code to the experimenter by email to signal completion and trigger payment. The online debrief, task instructions, and questionnaires were all delivered in each country’s official language, by local researchers.

### Statistical analyses:

All statistical analyses were performed and visualized using R^69,70,71^. The main dependent variable was the correct choice rate, i.e., choices directed toward the option with the highest expected value. Statistical effects were assessed by phase, using generalized linear mixed-effect models with a random intercept per participant^69^, with decision context and country of sample as categorical predictors (i.e. P(correct) ~ Decision Context × Country + ε , see ***Supplementary Materials*** for model selection). P-values were computed through Analysis of Deviance (Type II Wald χ^2^ test): we reported χ^2^, degrees of freedom and P-values. Proportion of variance explained per predictor was not reported because of how variance is partitioned in mixed models^72^. In cases where only one data point per participant was available (e.g. differences in parameter values across countries), statistical significance was evaluated through standard linear models using country as a categorical predictor (e.g., v_RL_ ~ Country). For those analyses, we reported F-statistic, Sum of Squares, P-value and Cohen’s F. Post-hoc contrasts were calculated with their respective confidence intervals, through estimated marginal means analysis, and P-values were Benjamini-Hochberg corrected. In particular, whenever we had to assess whether choice rate performances were significantly different from chance, we performed additional t-tests against chance level (0.5). In those cases, we reported the t-statistic, P-value, and Cohen’s *d* to estimate effect size. The significant association between continuous quantities (e.g. between parameter value and performance at a given decision context) was tested through correlation analysis, where we reported T-statistic, degrees of freedom, P-values, and *R*-coefficient as effect size. To prove lack of effect, we conducted AICc weight ratio analyses^73,74^ using a model containing the tested predictor (full) and its equivalent minus said predictor (null).

### Computational analyses:

the SCALING model was built around the notion of value scaling. Value scaling for both the RL and Lottery tasks was arbitrated by the free parameter (*v)* designed to capture value adaptation as follows:

$$R_{\text{scaled},t}=\{\begin{array}{ll} 10p*v,\;\text{if} & R_{obj,t}=10p\\ R_{obj,t} & \;\text{otherwise} \end{array}$$

where *R*_*scaled,t*_ represented the scaled objective reward *R*_*0bj,t*_ at trial *t*, and 0 ≤ *v* ≤ 1. For RL task trials, we used a simple Q-learning model^11^ to estimate in each choice context (or state) the expected reward (Q) of each option and pick the one that maximizes this expected reward Q. At trial *t*, option values (for example of the chosen option c) were updated according to the delta rule:

$$Q{\left(c\right)}_{t+1}=Q{\left(c\right)}_{t}+\alpha_{c}*\left(R{\left(c\right)}_{\text{scaled},t}-Q{\left(c\right)}_{t}\right)$$

where *α*_c_ is the learning rate for the chosen option, which, multiplied by the differecence between the *R*_*scaled*,t_ and Q_t_ is the prediction error term. We then modelled participants’ choice behaviour using a softmax decision rule that yielded the probability that for a state *s* a participant would choose, say, option *a* over option *b* according to:

$$P{\left(a\right)}_{t}=\frac{1}{1+e^{\beta *\left(Q{\left(b\right)}_{t}-Q{\left(a\right)}_{t}\right)}}$$

where β is the inverse temperature parameter. Low inverse temperatures (β → 0) cause the action to be stochastically equiprobable. High inverse temperatures (β → +∞) result in choices deterministically determined by the difference betwee the Q-values^11^. Our algorithm also included a forgetfulness parameter *ϕ* (0 ≤ *ϕ* ≤ 1) that allowed us to account for the possibility of forgetting the option values when moving from the Learning to the Transfer phases of the RL task. The Q-values used to fit (and simulate) the Transfer phase choices *(Q(:)*_*TRA*_) were calculated from the Q-values of the Learning phase *Q(: )*_*LEA*_ as follows:

$$Q{\left(:\right)}_{TRA}=Q{\left(:\right)}_{LEA}*\phi$$

For Lottery task, expected utilities *EU* of individual lotteries were calculated based on the described probability (*p*) its non-zero outcome and the subjective rescaled rewards *(R*_*scaied,t*_, calculated as for the Learning task). For example the expected value of lottery *a* was calculated as follows:

$$EU(a)=R{\left(a\right)}_{\text{scaled},t}*p(a)$$

Choice probabilities were also instantiated through a softmax rule, as follows (probability of choosing lottery *a*, over lotter *b*):

$$P{\left(a\right)}_{t}=\frac{1}{1+e^{\beta *(EU(b)-EU(a))}}$$

Since the lottery task does not involve learning or memory processes, its model lacked any notion of learning rate and forgetting parameter. The RL and the Lottery model shared the scaling parameter and the inverse temperature that were fitted specifically for each task *(v*_*RL*_ and *v*_*L0T*_; *β*_*RL*_ and *β*_*L0T*_).

Model parameters were fitted using maximum likelihood estimation using gradient descent as implemented in Matlab. Finally, in the ***Supplementary Materials* - Alternative Models** section we compared SCALING to three alternative computational models to discard other possible interpretations of our data. These included the ABSOLUTE model, which encoded outcomes on an absolute scale independently of the decision context in which they were presented; the ABSOLUTE-RISK model, which rescaled rewards for the RL task trials using the *v*_*lot*_ parameter fitted on Lottery task trials, in order to evaluate whether risk aversion predicted preference reversal; and the NEGLECT model, which assumed participants only learned the probabilities behind each choice, but ignored reward magnitude.

## Figures and Tables

**Table 1.** Demographic, sociocultural metrics and size of samples.

	USA	Israel	Japan	France	Chile	Argenti.	Russia	Iran	China	Morocco	India	ALL	P
**N (initial)**	51	58	55	58	59	51	58	60	53	56	64	623	--
**Exclusions**													
Completion issues	0	7	3	3	5	1	7	6	1	2	8	43	--
Rollout issues	1	1	2	1	0	0	1	5	3	3	2	19	--
**N (final)**	50	50	50	54	54	50	50	49	49	51	54	561	--
**Age (**mean(SD)**)**	26.5(4.2)	26(2.9)	20.6(1.7)	28.9(5.7)	22.5(2.2)	22.5(3.6)	26.3(4.1)	27(5.4)	23..4(2.8)	21.8(2.9)	23.1(4.9)	24.4(4.6)	<.0001
**Gender (**% fem.**)**	74	70	58	67	65	72	50	65	49	47	53	60.9	.99
**University education (**%**)**	95*	100	100	100	100	100	100	100	100	100	100	--	
**Human development Index 2019 (HDI)**	0.926	0.919	0.919	0.901	0.851	0.845	0.824	0.783	0.761	0.686	0.645	--	
**Cultural distance**													
From USA	--	0.1060	0.1222	0.1195	0.0627	0.0638	0.1369	0.0959	0.1618	0.1573	0.0845	--	--
From India	0.0845	0.1454	0.12	0.2811	0.0491	0.0525	0.0814	0.0669	0.1474	0.0975	--	--	--
**Socioeconomic Status (**mean(SD)**)**													
Childhood	3.9(0.3)	4.8(0.3)	6.1(0.2)	4.8(0.2)	5.9(0.3)	6.1(0.2)	4.3(0.3)	5.1(0.3)	4.2(0.3)	4.6(0.3)	5.2(0.3)	--	<.0001
Adulthood	3.9(0.3)	3.5(0.2)	5.7(0.3)	3.9(0.3)	4(0.2)	4.9(0.2)	4.2(0.2)	5.2(0.3)	4.8(0.3)	3.8(0.3)	5.1(0.3)	--	<.0001
Social hierarchy	5.4(0.3)	6.1(0.2)	7(0.2)	5.9(0.2)	6.7(0.2)	6.6(0.2)	5.5(0.2)	6.8(0.2)	5.2(0.3)	6.1(0.3)	6(0.3)	--	<.0001
**Individualistic & collectivistic tendencies (**mean(SD)**)**													
Vertical Ind.	18(0.9)	22(0.8)	23(0.8)	18(1)	17(1)	18(1)	21(0.7)	23(0.9)	26(0.8)	25(0.9)	24(0.7)	--	<.0001
Horizontal Ind.	29(0.6)	28(0.7)	25(0.8)	28(0.6)	29(0.6)	27(0.7)	26(0.7)	31(0.6)	28(0.8)	31(0.5)	28(0.8)	--	<.0001
Vertical Col.	24(1)	26(0.7)	21(0.9)	24(0.7)	25(0.9)	19(0.7)	19(0.7)	21(1)	27(0.7)	30(0.8)	30(0.9)	--	<.0001
Horizontal Col.	28(0.8)	28(0.8)	26(0.9)	27(0.6)	31(0.6)	31(0.5)	25(0.7)	25(0.7)	26(0.7)	30(0.7)	28(0.8)	--	<.0001
**Centrality of religiosity in social environment (**mean(SD)**)**													
Experiences	8(0.6)	6.8(0.5)	5.8(0.4)	6.8(0.5)	7.5(0.5)	5.7(0.4)	6.4(0.4)	9.1(0.5)	4(0.3)	13(0.4)	11(0.5)	--	<.0001
Role in ideology	9.9(0.6)	9(0.6)	8(0.4)	8.9(0.6)	10.5(0.4)	7.1(0.5)	8.3(0.6)	11(0.6)	5.3(0.4)	14(0.3)	11(0.5)	--	<.0001
Religious thought	7.6(0.4)	6.4(0.4)	7.7(0.3)	8.2(0.5)	6.6(0.4)	7.5(0.4)	7.3(0.4)	7.8(0.4)	5.8(0.4)	11(0.4)	9.1(0.5)	--	<.0001
Private life	7.8(0.4)	6(0.5)	7.3(0.4)	6.9(0.5)	7.6(0.5)	5.9(0.4)	6.1(0.4)	7.7(0.6)	5.4(0.4)	12(0.5)	10(0.5)	--	<.0001
Public life	5.6(0.5)	6.2(0.5)	5.7(0.3)	5.9(0.4)	5(0.4)	4.7(0.4)	4.4(0.3)	5.4(0.4)	4.1(0.3)	9.2(0.5)	8.6(0.5)	--	<.0001

* of the 78% of USA participants who chose to disclose their education level. P-values are Bonferroni-corrected for the number of comparisons presented in this table.
